# Supplementary material for: Structural adaptation of fungal cell wall in hypersaline environment
Source: Nat Commun. 2023 Nov 4;14:7082. doi: 10.1038/s41467-023-42693-6 (PMC10625518; doi:10.1038/s41467-023-42693-6)
Supplement: Supplementary file 1 — Supplementary Information [file 41467_2023_42693_MOESM1_ESM.pdf]

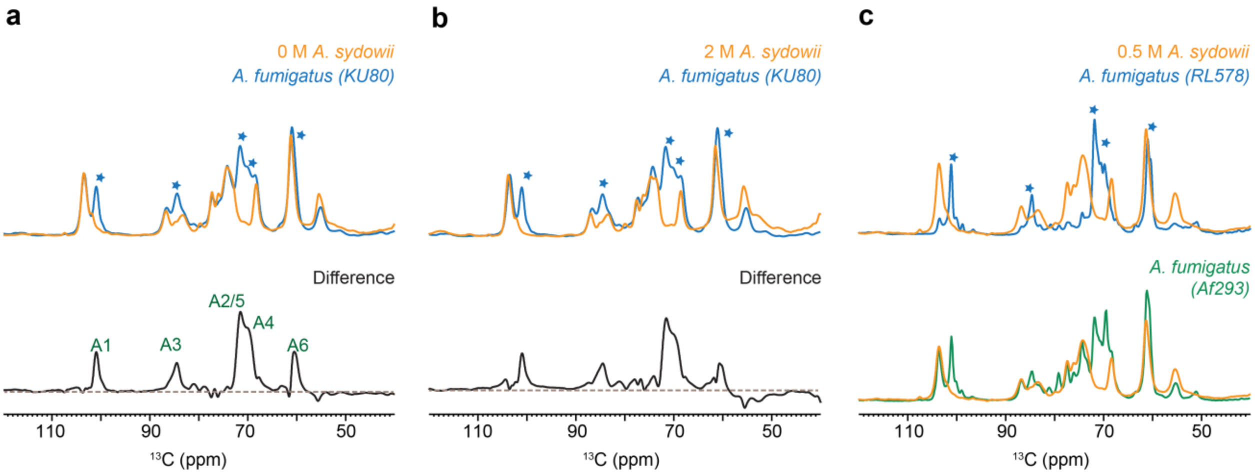

Supplementary Figure 1. Structural comparison of *A. sydowii* and *A. fumigatus*. Rigid polysaccharides shown by 1D ^{13}C CP spectra of *A. fumigatus* KU80 (cyan) cultured at 0.1 M NaCl and *A. sydowii* (yellow) cultured at **a**, 0 M NaCl **b**, 2.0 M NaCl. Asterisks indicate the positions where the peak intensities were low in the *A. sydowii* sample. Subtraction of two parental spectra generates a difference spectrum showing α-1,3-glucan (A) signals, which are missing in *A. sydowii*. **c**, Overlay of 0.5 M NaCl *A. sydowii* with non-halophilic fungi *A. fumigatus* RL578 (top) and Af293 (bottom).

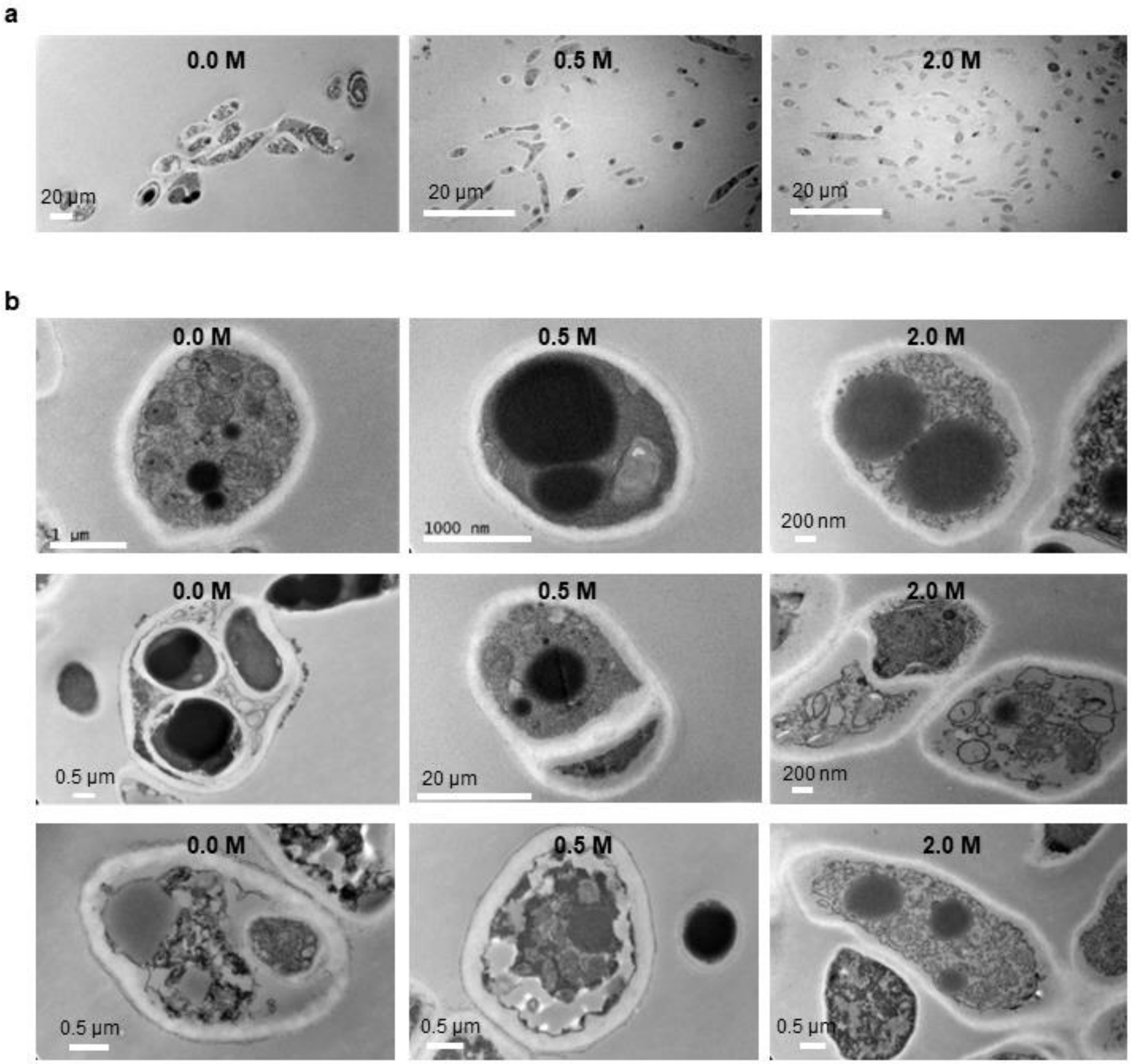

Supplementary Figure 2. Representative TEM images of *Aspergillus sydowii.* The images were taken from the perpendicular cross-sections of *A. sydowii* hyphae under **a,** low-magnification and **b,** high-magnification. The three columns of figures from the left to the right show the images of *A. sydowii* samples cultured at 0 M, 0.5 M, and 2 M NaCl conditions. White bars indicate the scale.

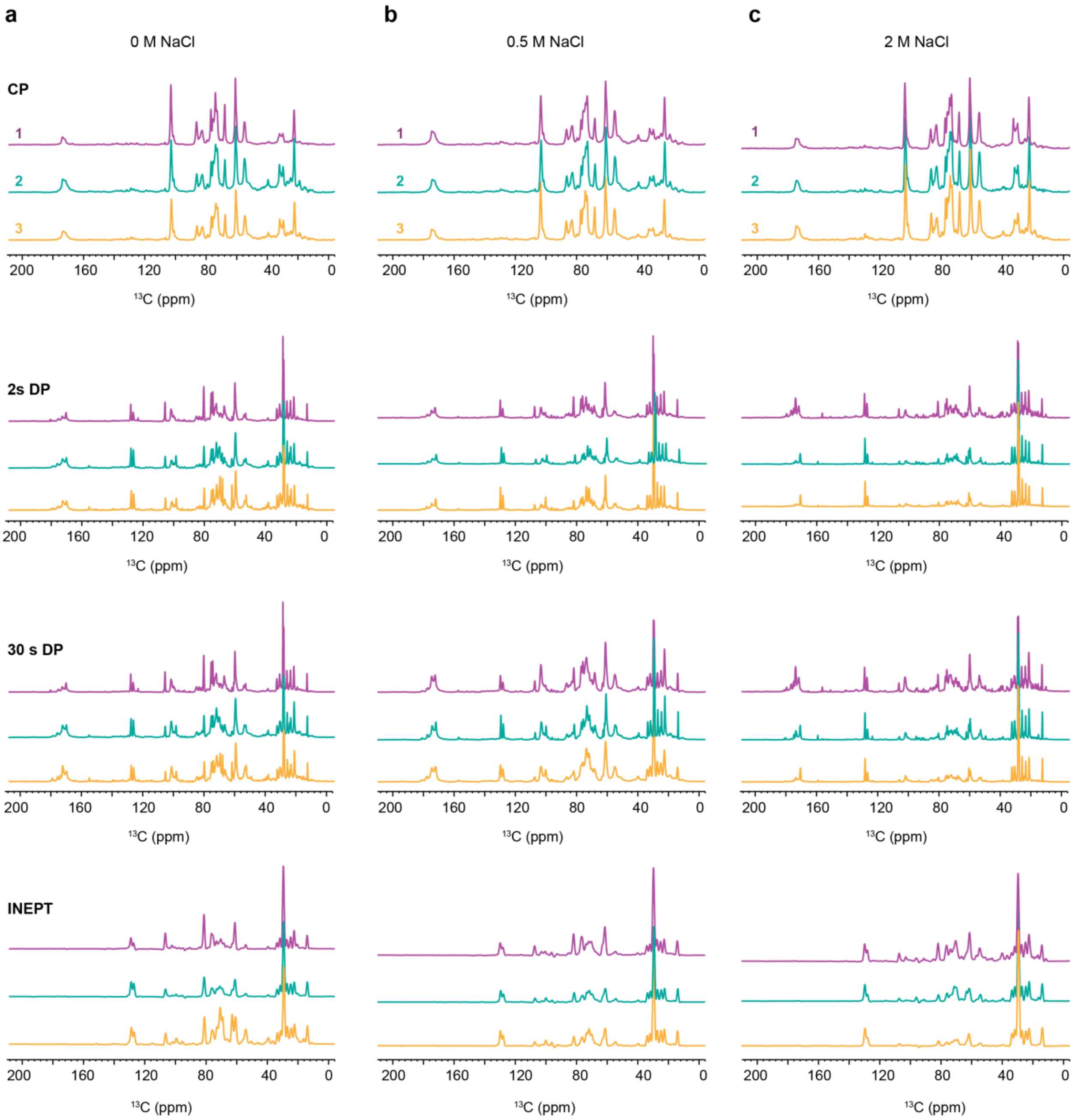

Supplementary Figure 3. Reproducibility of three batches of *A. sydowii* samples. 1D ^{13}C spectra were collected on three batches of halophilic fungi cultured in **a**, 0 M, **b,** 0.5 M , and **c**, 2 M NaCl. 1D ^{13}C CP spectra detecting the rigid molecules. 1D quantitative DP spectra were collected using a long recycle delay of 30 s for the unbiased detection of all molecules. 1D 2 s DP spectra detecting mobile molecules. 1D ^{13}C refocused INEPT. Three batches of replicates labeled as 1 (purple), 2 (green), and 3 (yellow). Samples were measured in 800 MHz spectrometer at 13 kHz MAS at 298 K.

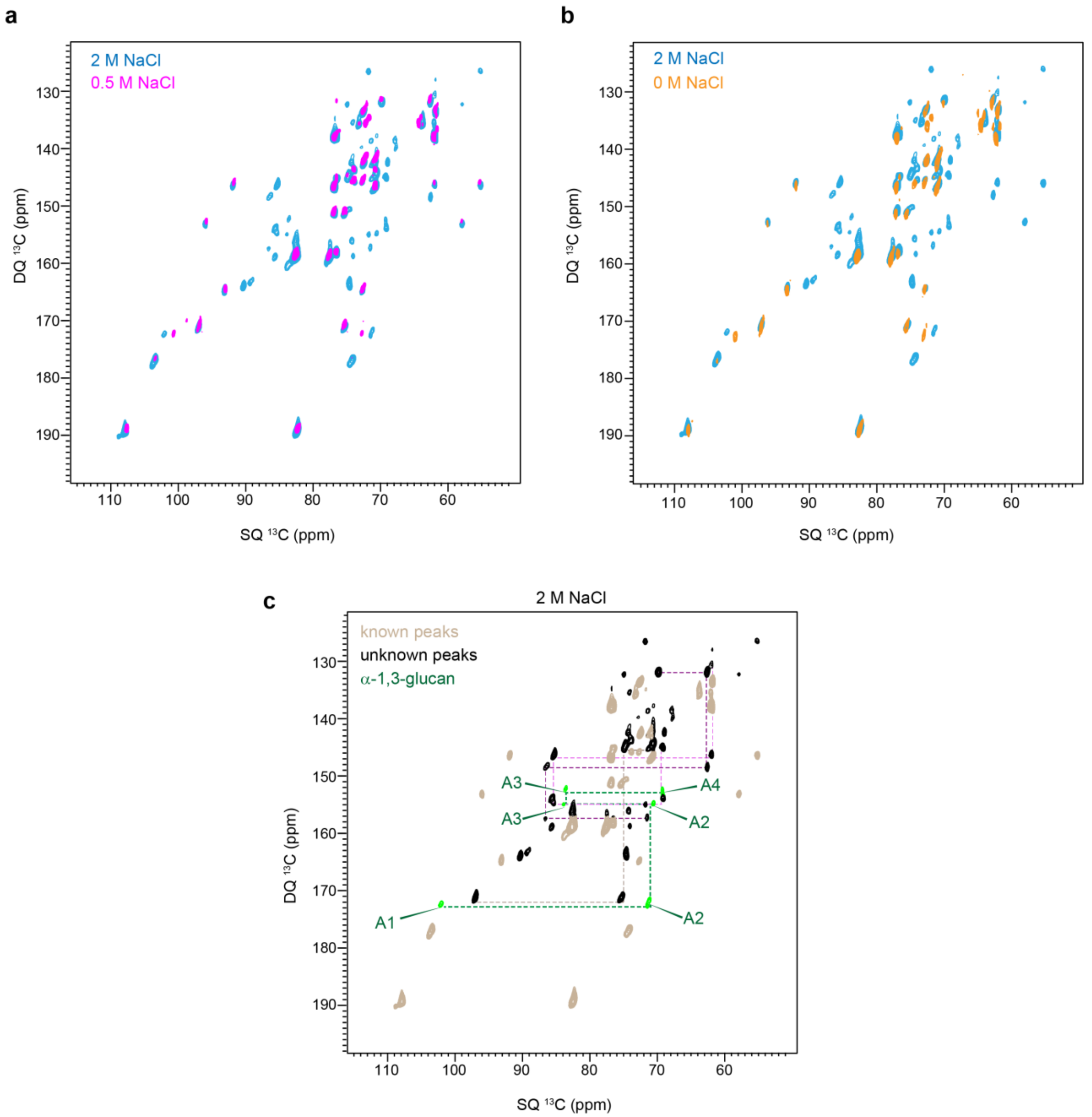

Supplementary Figure 4. Changes in mobile carbohydrate in response to high salinity. a, Overlay of 2D ^{13}C DP J-INADEQUATE spectra of *A. sydowii* samples cultured in 0.5 M (magenta) and 2 M (cyan) NaCl. **b,** Overlay of 2D ^{13}C DP J-INADEQUATE spectra of *A. sydowii* samples cultured in 0 M (orange) and 2 M (cyan) NaCl. **c,** 2D ^{13}C DP J-INADEQUATE spectra of 2 M NaCl showing the new peaks and α-1,3-glucan peaks. All spectra were measured in 850 MHz at 13 kHz MAS.

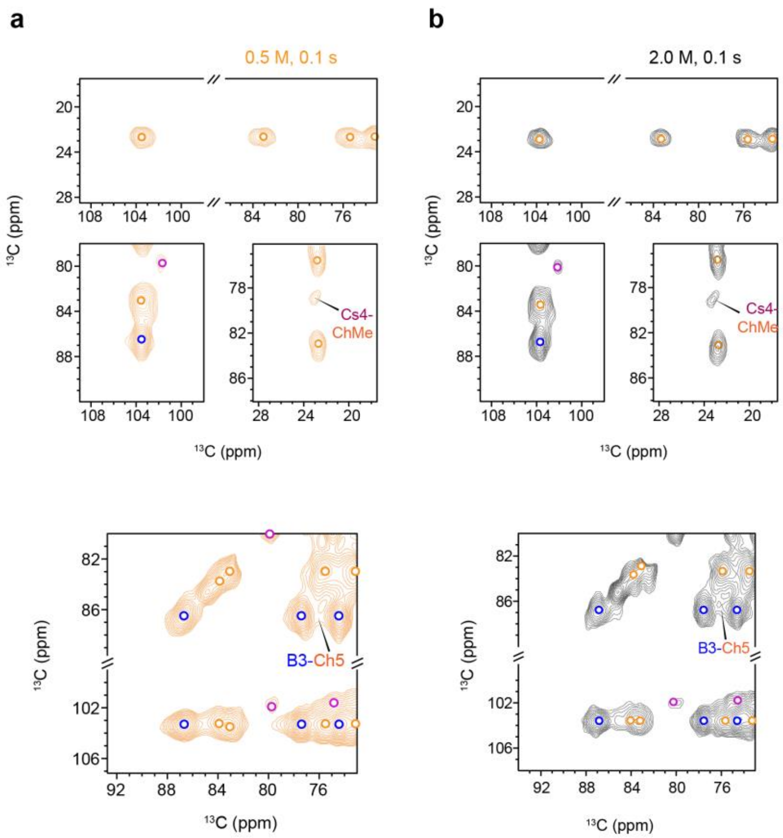

Supplementary Figure 5. Intermolecular contacts between *A. sydowii* polysaccharides. Selected regions were compared between **a,** 0.1 s DARR spectrum of sample cultured at 0.5 M NaCl, **b,** 0.1 s DARR spectrum of sample cultured at 2.0 M NaCl. and Open circles indicate the positions of intramolecular signals within chitin (orange), β-glucan (blue), and chitosan (purple). Intermolecular interactions are labeled. For example, Cs4-ChMe represents the cross peak between chitin methyl carbon and chitosan carbon 4. All spectra were measured on an 850 MHz NMR spectrometer.

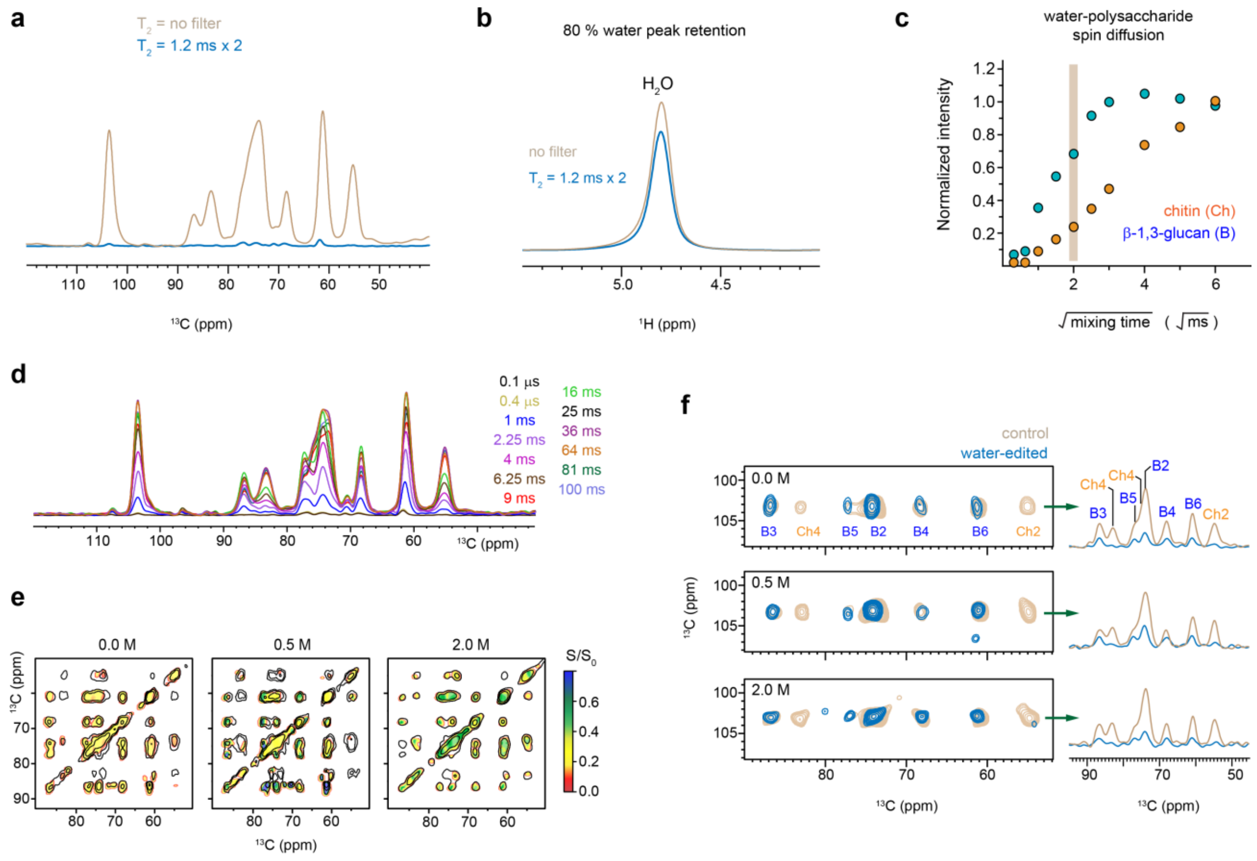

Supplementary Figure 6. Water-edited experiments for examining polymer hydration. a, Overlay of ^{13}C CP spectra of *A. sydowii* 0.5 M sample (in almond) with a ^{1}H T_2 filtered spectrum (T_2 = 1.2 ms x 2, blue). No spin diffusion was applied. 96% of carbohydrate signals were removed by the filter. **b**, 80% of water magnetization was retained after the ^{1}H-T_2 filter. **c**, Representative water-to-polysaccharide ^{1}H spin diffusion build-up curves. β-1,3-glucan has a faster buildup curve than chitin, revealing the hydrophilic nature of β-1,3 glucan. The curves are obtained from peak intensities of water-edited ^{13}C spectra. **d**, 1D water-edited ^{13}C spectra with different ^{1}H mixing times. **e**, Heatmap of relative water-edited intensity ratios (S/S_0) of *A. sydowii* cell wall polysaccharides presented as a 2D ^{13}C-^{13}C correlation spectra. **f**, Overlay of 2D water-edited spectra (blue) and control spectra (almond). The β-1,3-glucan peaks were selectively retained due to the water-association of this polysaccharide. 1D ^{13}C cross sections were extracted for comparison. All the spectra were measured on a 400 MHz spectrometer at 10 kHz MAS.

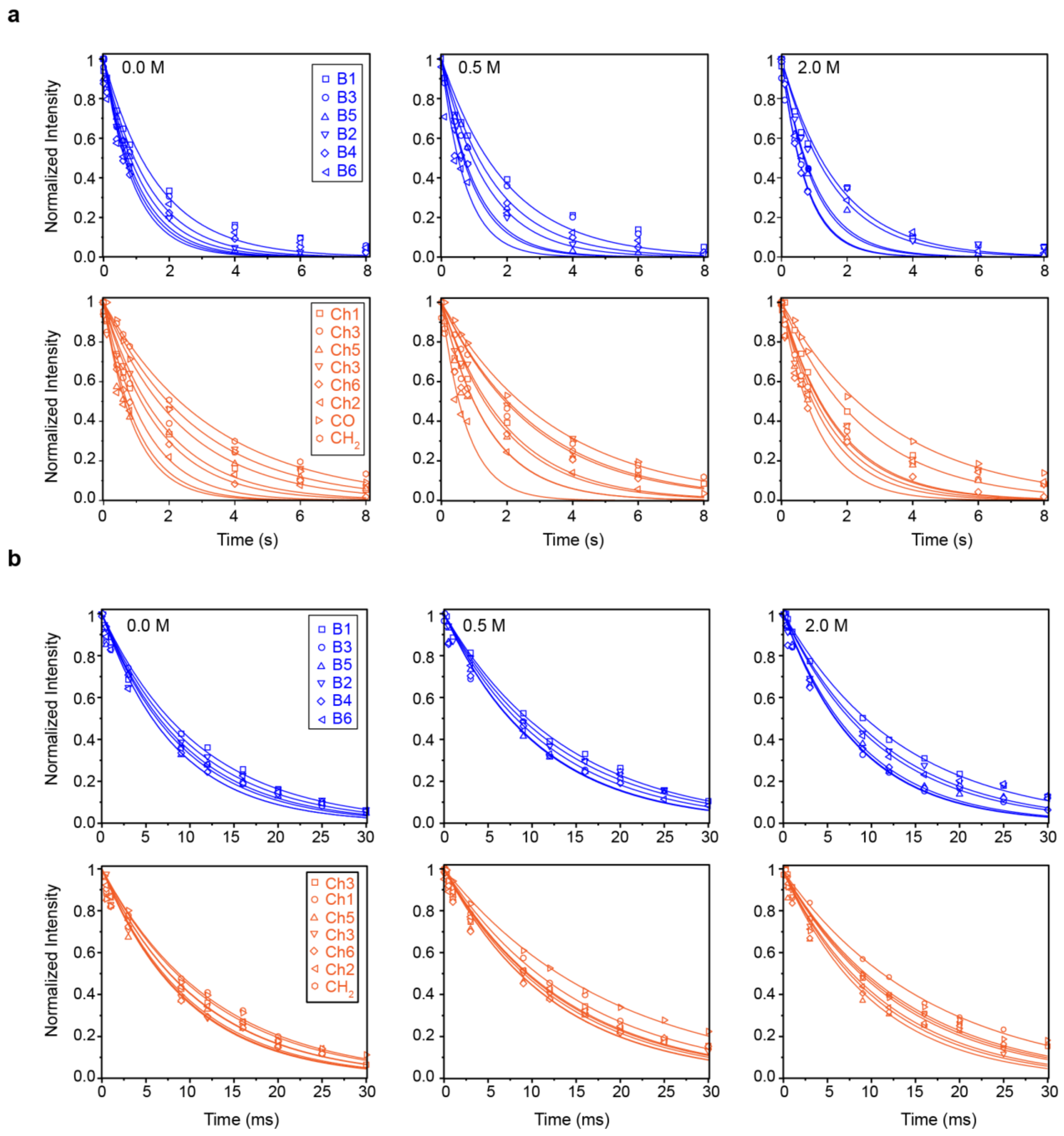

Supplementary Figure 7. NMR relaxation curves of polysaccharides. a, ^{13}C-T_1 and **b,** ^{1}H-$T_{1\rho}$ relaxation curves of polysaccharides in intact *A. sydowii* cell wall across 0 M, 0.5 M and 2 M NaCl. The data are collected on 400 MHz (9.4 Tesla) spectrometer at 10 kHz MAS and the best fit is achieved using single exponential equation. Blue curves are β-1,3-glucan and orange curves are chitin. Symbols are used for assigning different carbons in the polysaccharides.

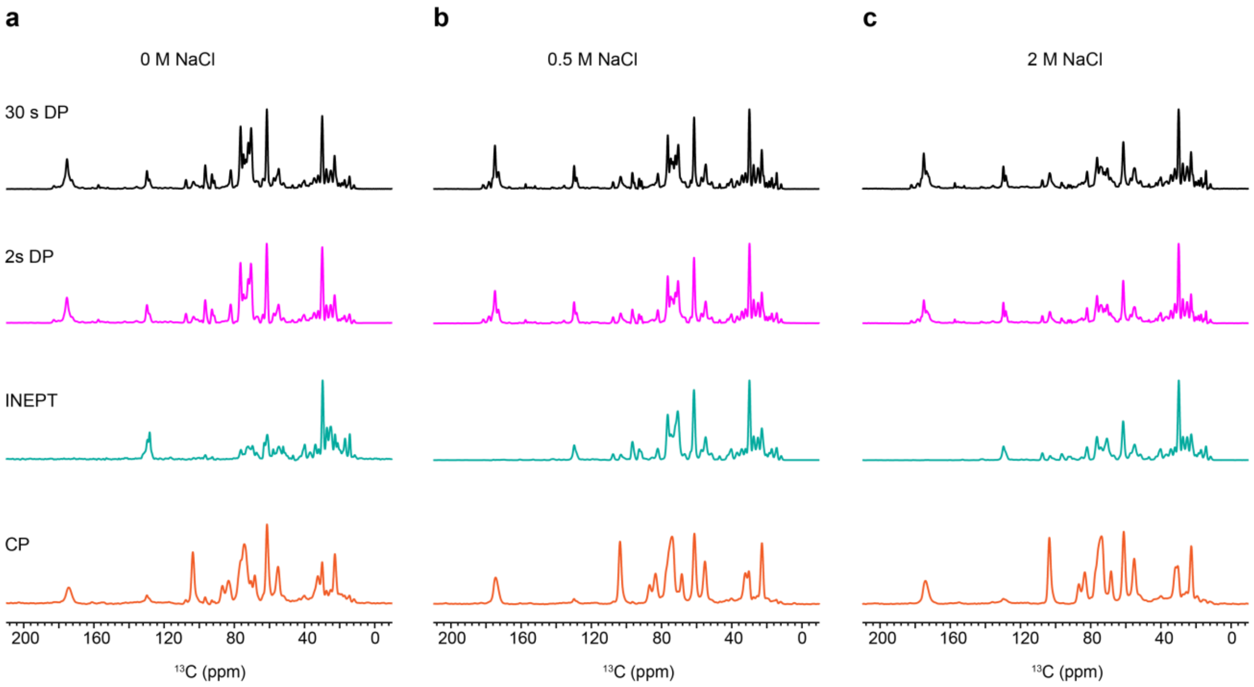

Supplementary Figure 8. ***A. sydowii*** **proteins and lipids mainly reside in the mobile phase.** An array of 1D ^{13}C spectra that detect different components with distinct dynamics are compared for *A. sydowii* samples cultured with **a,** 0 M, **b,** 0.5 M, and **c,** 2 M NaCl. These spectra include the 1D ^{13}C DP spectra measured with long recycle delays of 30 s (quantitative detection) and short recycle delays of 2 s (preferential detection of mobile molecules), 1D INEPT spectra (very mobile components) and 1D CP spectra (rigid molecules). All the spectra were measured on 400 MHz NMR under 10 kHz MAS.

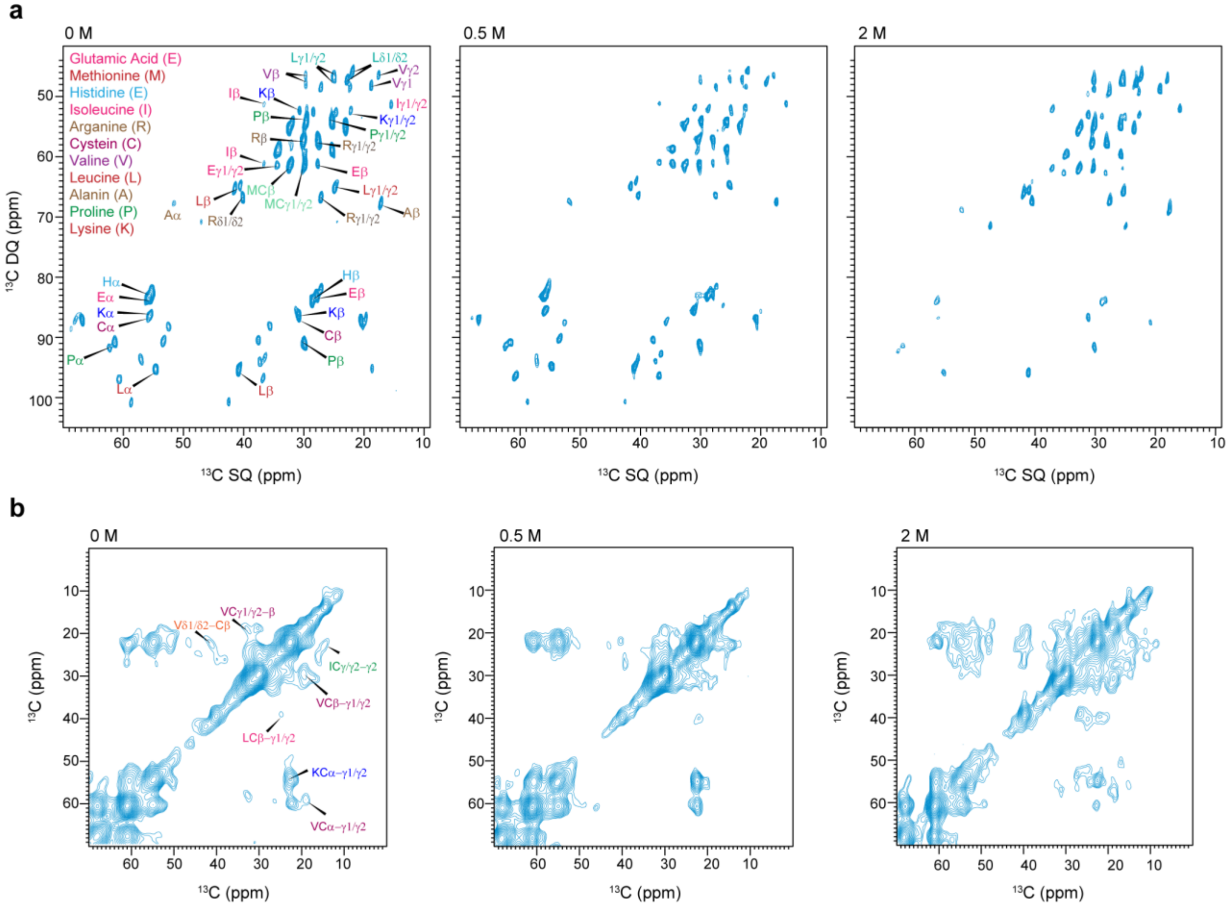

Supplementary Figure 9. Protein signals of mobile and rigid phases. a, 2D ^{13}C DP refocused J-INADEQUATE spectra showing signals of mobile proteins. **b,** 2D ^{13}C-^{13}C CP-based 100 ms DARR spectra showing the rigid proteins of 0 M, 0.5 M, and 2 M samples. All the refocused INADEQUATE spectra were measured on a 850 MHz spectrometer at 13 kHz MAS and all the DARR spectra were measured on a 400 MHz spectrometer at 10 kHz MAS.

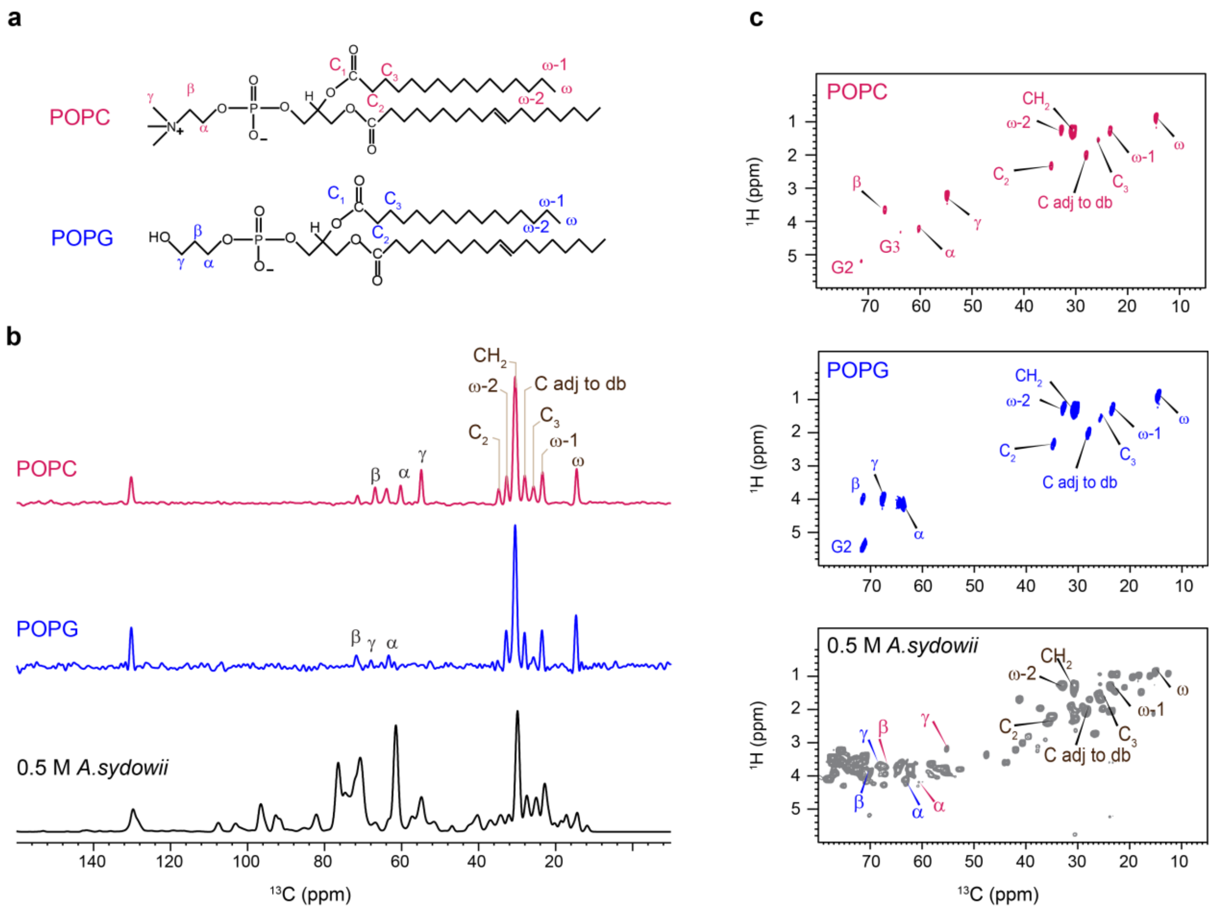

Supplementary Figure 10. 2D ^{1}H-^{13}C refocused INEPT spectra of phospholipids. a, Chemical structure of model phospholipids POPC and POPG with carbons labeled. **b,** 1D ^{13}C INEPT spectra and **c,** 2D ^{1}H-^{13}C spectra of POPC, POPG and *A. sydowii* with 0.5 M NaCl. The spectra were measured in 400 MHz spectrometer at 10 kHz MAS.

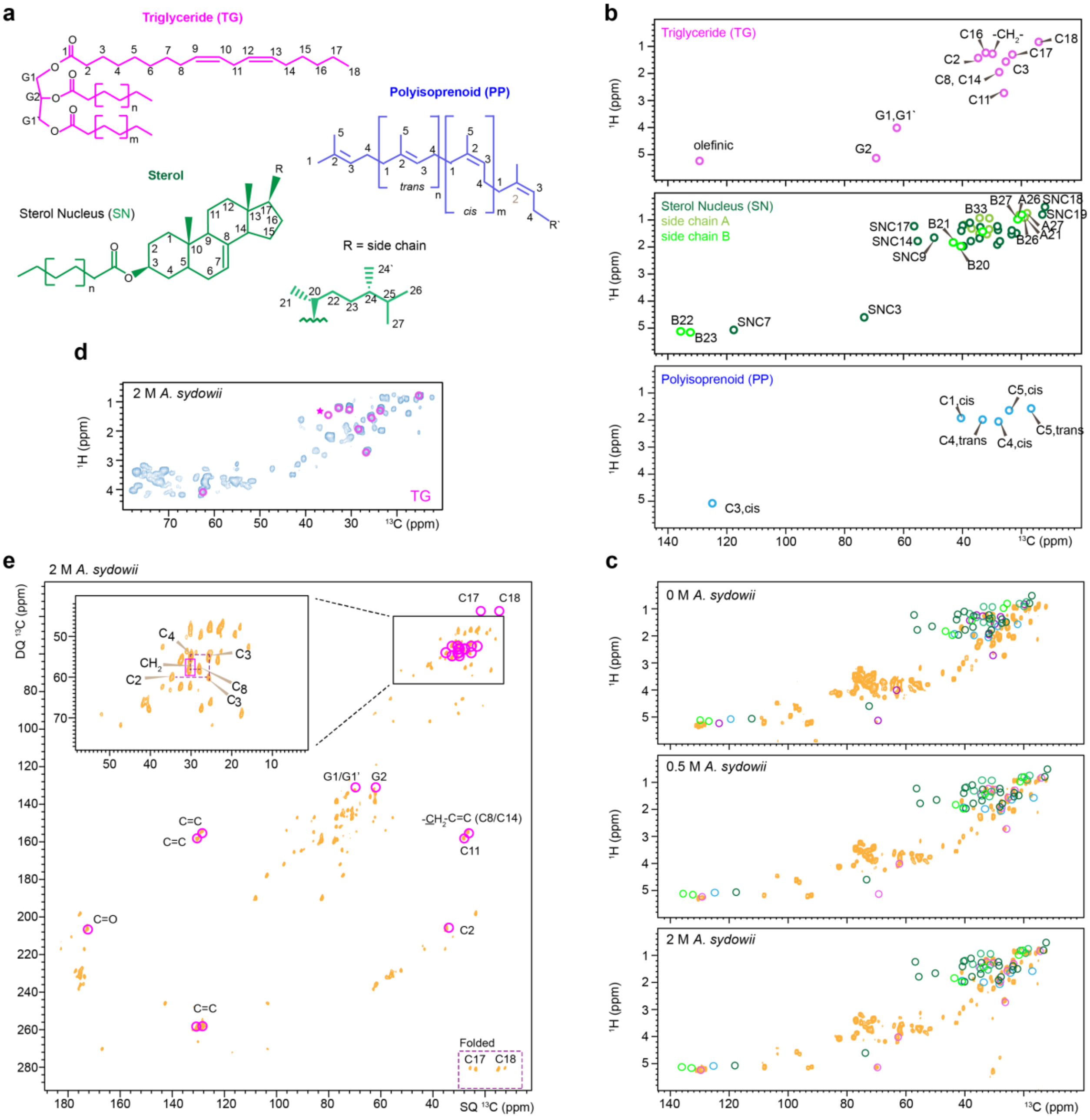

Supplementary Figure 11. Membrane and lipid components in *A. sydowii.* a, Representative structures of three lipid components: triglycerides (TG), Sterol (S), and polyisoprenoid (PP). **b,** Simulated 2D ^{1}H-^{13}C spectra using literature reported chemical shifts of TG, and S, and PP documented in **Supplementary Table 7**. **c,** Overlay of 2D ^{1}H-^{13}C refocused INEPT spectra of 0 M, 0.5 M and 2.0 M samples with stimulated spectra. The spectra of samples with 0 M and 0.5 M NaCl do not contain signals from triglycerides and sterols. **d,** Most TG signals are present in 2D ^{1}H-^{13}C refocused INEPT spectrum of *A. sydowii* with 2.0 M NaCl except for the signals of C2 (highlighted by asterisk). **e,** Overlay of simulated TG signals and experimentally measured DP refocused *J*-INADEQUATE spectrum of 2 M *A. sydowii*. All expected signals are present, though the signals are heavily overlapped with other lipids, amino acids, as well as the C5-C6 of some carbohydrates. Note that the signals of the C17-C18 spin pair is folded to the bottom right corner of the spectrum due to limited window width of the spectrum.

Supplementary Table 1. Media composition and culture condition of *Aspergillus* samples.

<table>
<tr><th>Samples</th><th>Media composition</th><th>[NaCl]</th><th>Temperature</th><th>rpm</th><th>Incubation time</th></tr>
<tr><td colspan="6">Halophiles</td></tr>
<tr><td>A. sydowii (0 M)</td><td rowspan="5">For 100 mL
Glucose (^{13}C) 2 g
$CuSO_4 \cdot 5H_2O$ 0.78 mg
$FeSO_4 \cdot 7H_2O$ 1.8 mg
$MgSO_4 \cdot 7H_2O$ 50 mg
$ZnSO_4$ 1 mg
KCl 5 mg
K_2HPO_4 0.1 g
NH_4NO_3 (^{15}N) 0.2 g
$CuSO_4 \cdot 5H_2O$ 0.78 mg
pH 6.0</td><td>0 M</td><td>28 °C</td><td>150</td><td>7 d</td></tr>
<tr><td>A. sydowii
(0.5 M)</td><td>0.5M</td><td>28 °C</td><td>150</td><td>7 d</td></tr>
<tr><td>A. sydowii
(2.0 M)</td><td>2.0 M</td><td>28 °C</td><td>150</td><td>7 d</td></tr>
<tr><td>A. atacamensis</td><td>1.0 M</td><td>28 °C</td><td>200</td><td>7 d</td></tr>
<tr><td>A. destruens</td><td>1.9 M</td><td>28 °C</td><td>200</td><td>7 d</td></tr>
<tr><td colspan="6">Aspergillus fumigatus</td></tr>
<tr><td>A. fumigatus Ku80</td><td>For 100 mL
Glucose (^{13}C) 1 g
$NaNO_3$ (^{15}N) 0.6 g
1000×TE 100 µL
50×Salt 2 mL
Mops 50mM
pH: 7.0</td><td>0.1 M</td><td>37°C</td><td>200</td><td>36 h</td></tr>
<tr><td>A. fumigatus
Af293</td><td>For 100 mL of Modified minimal media*
Glucose (^{13}C) 1.0 g
$NaNO_3$ (^{15}N) 0.6 g
KCl 0.052 g
KH_2PO_4 0.0815 g
K_2HPO_4 0.1045 g
$MgSO_4.7H_2O$ 0.052 g
$ZnSO_4.7H_2O$ 0.0022 g
H_3BO_3 0.0011 g
$MnCl_2.4H_2O$ 0.0005 g
$FeSO_4.7H_2O$ 0.0005 g
$CoCl_2.6H_2O$ 0.00016 g
$CuSO_4.5H_2O$ 0.00016 g
$(NH_4)_6Mo_7O_{24}.4H_2O$ 0.00011 g
$Na_4EDTA.4H_2O$ 0.006 g
pH 6.6</td><td>$[Na^+]$=0.07 M
$[Cl^-]$=0.007</td><td>30 °C</td><td>210</td><td>3 d</td></tr>
<tr><td>A. fumigatus
RL578</td><td>For 100 mL $^{13}C/^{15}N$ modified Czapek-Dox liquid medium
$NaNO_3$ 0.2 g
KCl 0.05 g
$C_3H_7MgO_6P$ 0.05 g
$FeSO_4$ 0.001 g
K_2SO_4 0.035 g
Sucrose 3 g
pH 7.4</td><td>$[Na^+]$=0.02 M
$[Cl^-]$=0.007</td><td>30 °C</td><td>0
(static)</td><td>14 d</td></tr>
</table>

Supplementary Table 2. Molar composition of polysaccharides in *A. sydowii* cell wall. The numbers were calculated using the integrals of well-resolved cross peaks of β-1,3 glucan and chitin in 2D ^{13}C-^{13}C DARR spectra. The results were already normalized by the number of scans. Error bars are standard errors of cross peak intensities. Not detected (-).

Rigid molecules				
Polysaccharide		0 M NaCl	0.5 M NaCl	2 M NaCl
β-1,3-glucan		55 ± 8%[a]	51 ± 9%[b]	40 ± 7%[c]
Chitin		33 ± 7 %[d]	39 ± 8%[e]	50 ± 13%[f]
Chitosan		12 ± 3%	10 ± 2%	10 ± 3%
**Mobile molecules**				
GAG	GalN	4 ± 1%	6 ± 2%	13 ± 3%
	GalNAc	4 ± 1%	5 ± 1%	9 ± 2%
	Gal*p*	20 ± 6%	24 ± 3%	14 ± 7%
GM		62 ± 13%	58 ± 15%	43 ± 17%
β-1,3-glucan		11 ± 3%	7 ± 2%	14 ± 5%
α-1,3-glucan		-	-	6 ± 1%

[a] Percentage taken from the average of B1-3, B1-5, B1-2, B1-4, B5-6, B3-4 cross peak integrations.
[b] The average of B1-3, B1-5, B1-2, B1-4, B3-5, B3-4 cross peak integrations.
[c] The average of B1-3, B1-5, B1-4, B3-2, B3-5, B3-4, B3-6, B5-4, B5-2 cross peak integrations.
[d] The average of Ch1-2, Ch1-6, Ch4-2, Ch5-2, Ch3-6, cross peak integrations.
[e] The average of Ch1-4, Ch1-6, Ch1-2, Ch4-6, Ch4-2, Ch5-2, cross peak integrations.
[f] The average of Ch1-4, Ch1-6, Ch1-3, Ch1-2, Ch4-2, Ch5-4, Ch5-2 cross peak integrations.

Supplementary Table 3. Intermolecular interactions identified by ssNMR. The table documented the cross peaks between different polysaccharides in *A. sydowii* samples cultured at 0.5 M and 2 M salinity conditions. The chemical shifts for the two dimensions of the spectra (ω_1 and ω_2), the assignment of the cross peak, the type of spectra, and the sample condition are summarized.

Cross peak	ω_1, ω_2 (ppm, ppm)	0.5 M 0.1 s PDSD	0.5M 1.5 s PDSD	ω_1, ω_2 (ppm, ppm)	2M 0.1 s PDSD	2M 1.5 s PDSD
Ch4-Ch4’	82.3, 84.1		x	82.3, 84.1		x
Ch4’-Ch4	84.1, 82.3		x	84.1, 82.3		x
ChMe-B5	23.1, 77.1		x	23.1, 77.1		x
B3-Ch5	86.1, 76.1	x	x	86.1, 76.1	x	x
B5-Ch5	68.2, 75.5	x	x	68.5, 75.8	x	x
ChMe-Cs4	22.9, 79.4		x	22.7, 79.9		x
ChMe-Cs1	22.9, 101.5		x	22.7, 102.0		x
Ch4-Cs1	83.5, 101.5		x			
Ch1/B1-Cs4	103.2, 79.5		x			
Cs4-ChMe	79.2, 22.9	x	x	79.2, 22.8	x	x
Cs4-ChCO	79.7, 173.6			79.7, 173.8		x
Cs1-Ch4	102.1, 83.1		x			
B1-Cs1				86.8, 102.1		x
B3-Cs4	86.5, 79.2		x			
B5-Cs4	68.2, 79.2		x			
Cs1-B5	101.5, 77.2		x			

Supplementary Table 4. Water-edited intensities of polysaccharides cross peaks. The intensity ratios were obtained by comparing the peak intensity in water-edited and control spectra, with normalization by the number of scans. Error bars are standard deviations propagated from NMR signal-to-noise ratios.

	**0.5 M NaCl**		**0 M NaCl**		**2 M NaCl**	
	**Cross peak**	**Intensity**	**Cross peak**	**Intensity**	**Cross peak**	**Intensity**
β-1,3-glucan	B1-3	0.5±0.1	B1-3	0.42±0.09	B1-3	0.4±0.1
	B1-5	0.5±0.1	B1-5	0.37±0.09	B1-5	0.5±0.1
	B1-2	0.41±0.04	B1-2	0.26±0.03	B1-2	0.31±0.04
	B1-4	0.5±0.1	B1-4	0.29±0.07	B1-4	0.5±0.1
	B1-6	0.39±0.07	B1-6	0.28±0.06	B1-6	0.34±0.09
	B3-1	0.7±0.1	B3-1	0.43±0.07	B3-1	0.5±0.1
	B3-2	0.61±0.09	B3-5	0.34±0.06	B3-5	0.4±0.1
	B3-4	0.5±0.1	B3-2	0.44±0.06	B3-2	0.3±0.1
	B3-6	0.80±0.07	B3-4	0.38±0.07	B3-4	0.4±0.1
	B5-1	0.45±0.08	B3-6	0.59±0.06	B3-6	0.5±0.2
	B5-3	0.7±0.2	B5-1	0.35±0.07	B5-1	0.4±0.1
	B5-2	0.40±0.07	B5-3	0.4±0.1	B5-3	0.5±0.2
	B5-4	0.44±0.08	B5-2	0.25±0.07	B5-2	0.21±0.07
	B5-6	0.39±0.05	B5-4	0.28±0.06	B5-4	0.36±0.07
	B2-1	0.39±0.04	B5-6	0.30±0.05	B5-6	0.37±0.06
	B2-3	0.6±0.1	B2-1	0.29±0.03	B2-1	0.25±0.04
	B2-5	0.7±0.1	B2-3	0.39±0.09	B2-3	0.6±0.2
	B2-4	0.6±0.1	B2-5	0.53±0.09	B2-5	0.5±0.1
	B2-6	0.31±0.04	B2-4	0.36±0.07	B2-4	0.38±0.07
	B4-1	0.6±0.1	B2-6	0.25±0.04	B2-6	0.26±0.05
	B4-3	0.6±0.1	B4-1	0.36±0.07	B4-1	0.5±0.1
	B4-5	0.6±0.1	B4-3	0.37±0.09	B4-3	0.3±0.1
	B4-2	0.43±0.09	B4-5	0.38±0.07	B4-5	0.4±0.1
	B4-6	0.5±0.1	B4-2	0.42±0.08	B4-2	0.28±0.08
			B4-6	0.26±0.07	B4-6	0.33±0.09
Chitin	Ch1-3	0.13±0.02	Ch1-4	0.21±0.02	Ch1-4	0.14±0.09
	Ch1-6	0.38±0.07	Ch1-5	0.38±0.05	Ch1-3	0.23±0.04
	Ch4-1	0.12±0.09	Ch1-2	0.12±0.06	Ch4-1	0.2±0.1
	Ch4-4	0.23±0.08	Ch4-4	0.22±0.08	Ch5-1	0.27±0.08
	Ch5-1	0.35±0.06	Ch5-1	0.42±0.06	Ch5-3	0.06±0.04
	Ch5-4	0.15±0.8	Ch5-4	0.17±0.09	Ch5-6	0.19±0.5
	Ch5-3	0.2±0.03	Ch5-3	0.21±0.05	Ch5-2	0.2±0.1
	Ch5-6	0.27±0.05	Ch5-6	0.31±0.04	Ch3-1	0.26±0.07
	Ch3-1	0.22±0.05	Ch5-2	0.09±0.06	Ch3-4	0.1±0.1
	Ch3-5	0.12±0.01	Ch3-1	0.31±0.05	Ch3-6	0.29±0.09
	Ch3-6	0.21±0.07	Ch3-5	0.29±0.02	Ch3-2	0.14±0.08
	Ch3-2	0.13±0.07	Ch3-6	0.22±0.06	Ch2-1	0.2±0.1
	Ch2-1	0.22±0.07	Ch3-2	0.15±0.05	Ch2-6	0.2±0.1
	Ch2-4	0.2±0.1	Ch2-1	0.17±0.09	Ch2-2	0.1±0.04
	Ch2-5	0.12±0.07	Ch2-3	0.08±0.07		
	Ch2-3	0.13±0.06				
	Ch2-6	0.2±0.1				

Supplementary Table 5. ^{13}C-T_1 and ^{1}H-$T_{1\rho}$ relaxation time constants of polysaccharides in *A. sydowii*. A single exponential equation was used to fit the T_1 data $I(t) = e^{-t/T_I}$. A single exponential equation was used to fit the $T_{1\rho}$ data: $I(t) = e^{-t/T_{I\rho}}$. Error bars are standard deviations of the fit parameters.

Sample Type	Cross peaks	T_1 (s)	Cross peaks	$T_{1\rho}$ (ms)
0.5 M NaCl	B3	1.5±0.1	B1	13.8±0.8
	B5	0.8±0.2	B3	11±1
	B2	1.6±0.3	B5	11±1
	B4	0.7±0.3	B2	12.7±0.9
	B6	0.5±0.1	B4	11±1
	Ch1	2.2±0.2	B6	12±1
	Ch5	1.0±0.2	Ch1	13.8±0.8
	Ch3	2.2±0.4	Ch4	15.3±0.8
	Ch6	0.8±0.1	Ch5	12.4±0.9
	Ch2	2.0±0.4	Ch3	13.1±0.8
			Ch6	12±1
			Ch2	13.5±0.8
0 M NaCl	B1	1.7 ±0.2	B1	11±0.6
	B3	1.28±0.07	B3	8.9±0.9
	B5	0.9±0.1	B5	8.3±0.8
	B2	1.4±0.2	B2	10.1±0.6
	B4	0.88±0.05	B4	8.3±0.8
	B6	0.9±0.1	B6	9.4±0.6
	Ch1	1.7±0.2	Ch1	11.0±0.6
	Ch4	2.9±0.3	Ch4	12.6±0.9
	Ch5	1.1±0.2	Ch5	9.8±0.9
	Ch3	1.3±0.2	Ch3	10.1±0.7
	Ch2	1.5±0.3	Ch6	9.7±0.6
			Ch2	11.1±0.6
2.0 M NaCl	B1	1.7±0.2	B1	13.5±0.6
	B3	0.77±0.07	B3	8.6±0.7
	B5	0.98±0.09	B5	8.9±0.7
	B2	1.6±0.2	B6	11±1
	B6	1.0±0.1	B2	11.6±0.9
	Ch1	1.7±0.2	Ch4	8.5±0.9
	Ch4	2.4±0.4	Ch5	10.9±0.8
	Ch5	1.3±0.2	Ch6	11.2±0.9
	Ch3	1.7±0.3	Ch2	11.7±0.8
	Ch2	1.5±0.2	Ch3	13.0±0.9

Supplementary Table 6. Water-edited intensities of amino acid residues. The intensity ratios are obtained by comparing the peak intensity in 1D ^{13}C water-edited and control spectra, with normalization by the number of scans. Error bars are standard deviations propagated from NMR signal-to-noise ratio.

0.5 M NaCl		0 M NaCl		2 M NaCl	
^{13}C (ppm)	Intensity	^{13}C (ppm)	Intensity	^{13}C (ppm)	Intensity
52.0	0.14± 0.09	52.0	0.2 ± 0.1	52.0	0.17 ± 0.07
43.2	0.12 ± 0.09	46.9	0.3 ± 0.1	43.2	0.15 ± 0.07
40.3	0.20 ± 0.09	43.2	0.2 ± 0.1	40.3	0.22 ± 0.07
32.6	0.11 ± 0.09	40.3	0.3 ± 0.1	32.6	0.14 ± 0.07
30.1	0.37 ± 0.09	30.1	0.5 ± 0.1	30.1	0.28 ± 0.07
27.6	0.27 ± 0.09	27.6	0.3 ± 0.0.1	27.6	0.16 ± 0.07
25.2	0.27 ± 0.09	25.2	0.4 ± 0.1	25.2	0.15 ± 0.07
19.2	0.16 ± 0.09	22.8	0.31 ± 0.1	19.2	0.14 ± 0.07
17.3	0.36± 0.09	19.2	0.2 ± 0.1	14.6	0.29 ± 0.07
14.6	0.34 ± 0.09	17.3	0.6 ± 0.1	12.0	0.11 ± 0.07
12.0	0.20± 0.09	14.6	0.6 ± 0.1		
		12.0	0.4 ± 0.1		

Supplementary Table 7. Chemical shifts of lipids. Chemical shifts of PC and PG lipids are from measurements. The other components were from literature. Not applicable (/). Unidentified (-).

Lipid	Carbon	^{13}C (ppm)	^{1}H (ppm)	Reference
PC	C2	34.8	2.3	
	C3	25.7	1.6	
	-CH2	30.5	1.31	
	$\omega - 2$	32.7	1.3	
	$\omega - 1$	23.4	1.3	
	ω	14.02	0.9	
	α	60.3	4.3	
	β	66.8	3.6	
	γ	54.7	3.2	
	G1	63.5	-	
	G2	71.4	4.4	
	G3	63.8	4.0	
Triglycerides	C1	172.4	/	Chrissian et al. 2020[1] Lamon et al. 2023[2]
	C2	34.3	1.44	
	C3	25.0	1.57	
	-(CH2)$_n$-	29.3-29.9	1.26-1.29	
	C8,C14	27.3	1.96	
	C9-C10,C12,13	128.1-130.0	5.26-5.29	
	C11	25.6	2.74	
	C16	32.0	1.25	
	C17	22.8	1.29	
	C18	14.1	0.86	
	G1,G1’	62.2	4.04 (α) 4.25 (β)	
	G2	69.1	5.18	
Sterol side chain A	C20	36.9	1.33	Suttiarporn et al. 2015[4] Chrissian et al 2020[1]
	C21	19.1	0.93	
	C22	34.2	0.93 (α), 1.49 (β)	
	C23	30.9	0.93 (α), 1.37 (β)	
	C24	39.2	1.2	
	C25	31.7	1.55	
	C26	20.7	0.81	
	C27	18.1	0.77	
Sterol side chain B	C20	40.6	1.98	Tuckey et al. 2012[5]
	C21	21.3	1.00	
	C22	135.6	5.15	
	C23	132.0	5.20	
	C24	43.1	1.83	
	C25	33.3	1.44	
	C26	19.9	0.81	
	C27	19.9	0.81	

Lipid	Carbon	^{13}C (ppm)	^{1}H (ppm)	Reference
PG	C2	34.8	2.3	
	C3	25.7	1.6	
	-CH2	30.5	1.31	
	$\omega - 2$	32.7	1.3	
	$\omega - 1$	23.4	1.3	
	ω	14.02	0.9	
	α	63.4	4.1	
	β	71.1	4.0	
	γ	68.1	3.9	
	G1	-	-	
	G2	71.4	5.3	
	G3	-	-	
Polyisoprenoids	C1 trans	39.9	1.94	Chrissian et al 2020[1]
	C1, trans	134.4	/	
	C3 trans	124.4	5.07	
	C4, trans	27.1	2.06	
	C5, trans	16.0	1.56	
	C1, cis	32.4	1.99	
	C2, cis	135.0	/	
	C3, cis	124.8	5.07	
	C4, cis	26.7	1.99	
	C5, cis	23.5	1.63	
Δ^7- sterol nucleus	C1	37.1	1.13(α), 1.79 (β)	Ragasa et al. 2012[3] Chrissian et al. 2020[1]
	C2	27.7	1.79 (α), 1.39 (β)	
	C3	73.1	4.62	
	C4	34.1	1.69 (α), 1.28 (β)	
	C5	40.2	1.42	
	C6	29.7	-	
	C7	117.6	5.12	
	C8	139.7	/	
	C9	49.5	1.66	
	C10	34.2	/	
	C11	21.7	1.54 (α), 1.48(β)	
	C12	39.8	1.21 (α), 1.97 (β)	
	C13	43.3	/	
	C14	55.1	1.80	
	C15	23.2	1.55 (α), 1.97 (β)	
	C16	28.1	1.89 (α), 1.26 (β)	
	C17	56.3	1.23	
	C18	11.9	0.53	
	C19	12.9	0.79	

Supplementary Table 8. **Recipe of mineral-base liquid medium.** The pH is adjusted to 6.0 with H_3PO_4 or 0.25 M KOH. Each sample uses 100 mL of medium that contains 2 g of ^{13}C- glucose and 0.2 g $^{15}NH_4NO_3$. The culture media and condition were adapted from a previously described protocol[6].

Reagent	For 1 liter
$CuSO_4 \cdot 5H_2O$	7.8 mg
$FeSO_4 \cdot 7H_2O$	18 mg
$MgSO_4 \cdot 7H_2O$	500 mg
$ZnSO_4$	10 mg
KCl	50 mg
K_2HPO_4	1 g
$^{15}NH_4NO_3$	2 g
$CuSO_4 \cdot 5H_2O$	7.8 mg

Supplementary Table 9. Solid-state NMR experiments and parameters for each of the three *A. Sydowii* samples. To be quantitative, direct pulse (DP) experiments with 30 s long recycling delay were used. cross-polarization (CP), most rigid molecules. With DP and a shorter recycling delay of 2 seconds, suppress the rigid molecules from the spectra, and with Insensitive Nuclei Enhanced by Polarization Transfer (INEPT) the most mobile molecules were selected. For 2D ^{13}C-^{13}C correlation experiments, DARR (Dipolar Assisted Rotational Resonance) allowed to resolve rigid intramolecular peaks. While 1.5 s PDSD (Proton Driven Spin Diffusion) detects intra- and inter-molecular peaks. 2D DQ-SQ, DP J-INADEQAUTE and CP INADEQUATE spectra were used to detect through-bond correlations. The experimental parameters include the ^{1}H Larmor frequency, total experiment time (t), recycle delay (d1), number of scans (NS), The number of points for the direct (td2) and indirect (td1) dimensions, the acquisition time of the direct dimension (aq2) and the evolution time of indirect dimension (aq1), spectral width (sw1 and sw2), mixing time (t_m), increment delay (IN_F) and T filter times. * Indicates the water-polysaccharide spin diffusion and the DARR mixing time. The processing parameters include the window function and associated parameters.

	Acquisition parameters													Processing parameters	
Experiment	$\omega_{0,\,1H}$ (MHz)	t (h)	d1 (s)	NS	td2	td1	aq2 (ms)	aq1 (ms)	sw2 (ppm)	sw1 (ppm)	t_m (ms)	IN_F (µs)	T filters	Window function	Parameter
1D CP	850	0.1	2	256	7360		14.7		1169					QSINE	SSB 3
1D DP	850	0.1	2	256	7360		14.7		1169					QSINE	SSB 3
1D DP	850	2.1	30	256	7360		14.7		1169					QSINE	SSB 3
1D INEPT	400	0.1	3	64	3600		36.0		496.6					GM	LB-5, GB0.01
1D ^{13}C T1	400	3.5	2	256	1600		16.0		496.6				T_1(10^{-3}-8 s)		
1D ^{1}H T1ρ	400		2	256	1400		14.0		496.6				SL (10^{-3}-30 ms)		
2D DARR	850	4.2	2	48	1472	512	14.7	5.1	233.9	233.9	100	20		QSINE	SSB 3
2D PDSD	850	13.6	2	32	2496	512	24.9	5.1	233.9	233.9	1500	20		QSINE	SSB 2.8
2D DP INADEQUATE	850	4.5	2	32	7360	256	14.7	2.5	1169	243.4		19.2		QSINE	SSB 3
Water edited - control	400	17	1.6	256	1400	152	14.0	4.9	496.6	152.8	10^{-4} /50*	65	T_2 10^{-4} ms	QSINE	SSB 4.5
Water edited	400	17	1.6	256	1400	152	14.0	4.9	496.6	152.8	4/50*	65	T_2 1.2 ms	QSINE	SSB 4.5
C-H INEPT	400		3.0	8	2048	160	20.0	11.0	496.6	18.1		137		QSINE	SSB 3

Supplementary Table 10. ^{13}C and ^{15}N chemical shifts of *A. sydowii* polysaccharides and proteins. Superscripts are used to denote different allomorphs. Underline denotes the ^{13}C connectivity with ambiguity. Not applicable (/). Unidentified (-). Unknown: (Unk).

Polysaccharides	NMR abbreviation	C1 (ppm)	C2 (ppm)	C3 (ppm)	C4 (ppm)	C5 (ppm)	C6 (ppm)	CO (ppm)	CH_3 (ppm)	References
β-1,3-glucan	B	103.6	74.4	86.4	68.7	77.1	61.3	/	/	Shim *et al.* 2007[7]
Chitin	Ch^{a}	103.3	55.5	72.9	84.5	76.2	60.7	174.6	22.7	Fernando et al. 2021[8]
	Ch^{b}	103.5	55.2	73.4	84.4	75.9	60.0	173.4	22.7	
	Ch^{c}	103.5	55.4	73.3	83.7	75.9	60.7	173.4	22.7	
	Ch^{d}	103.6	55.0	73.2	83.4	75.5	60.5	174.1	22.4	
	Ch^{e}	103.2	54,8	73.5	82.5	75.2	60.9	175.1	22.4	
Chitosan	Cs^{a}	102.2	55.6	74.5	80.4	74.9	60.7	/	/	
	Cs^{b}	101.9	55.7	72.9	80.0	74.3	60.5	/	/	
	Cs^{c}	101.4	55.5	73.5	79.1	75.3	61.0	/	/	
	Cs^{d}	101.4	55.5	73.5	79.1	75.3	61.0	/	/	
α-1,3-glucan	A	101.0	71.9	84.6	69.5	71.7	60.5	/	/	Bhanja *et al.*2014[9]
α-1,4-galactan	Gal	93.2	72.2	70.7	73.5	72.5	60.9	/	/	Chakraborty et al 2021[10]
α-1,4-galactosamine	GalN	91.7	54.8	71.1	81.1	-	-	/	/	
α-1,4-N-acetylgalactosamine	GalNAc	95.7	57.5	75.2	76.9	-	-	-	-	
α-1,6-manose	$\mathrm{Mn}^{1,6}$	102.7	70.6	73.2	72.5	73.7	66.1	/	/	Chakraborty et al 2021[10]
α-1,2-manose	$\mathrm{Mn}^{1,2}$	101.3	78.7	71.2	67.7	73.9	61.7	/	/	
β-1,5 galactofuranose	Galf	107.5	81.5	77.7	83.5	71.5	63.4	/	/	
Unknown	Unk	102.6	84.9	73.5	68.5	-	-			
**Amino Acid**	**Abbreviation**	**Cα (ppm)**	**Cβ (ppm)**	**Cγ/γ1 (ppm)**	**Cγ2 (ppm)**	**Cδ/δ1 (ppm)**				
Glutamic Acid	E	55.8	27.8	34.4						Fritzsching. et al 2013[11]
Methionine	M		32.8	30.1						
Histidine	H	55.8	28.2							
Isoleucine	I		37.3	25.3	15.6					
Arginine	R			27.7		40.4				
Cysteine	C	55.8	31.0							
Valine	V		30.0	19.2						
Leucine	L			25.3		22.4				
Alanine	A	52.1	17.6							
Proline	P	61.7	30.1	25.8						
Lysine	K	55.2	41.3	40.8	25.0					

Supplementary References

1. Chrissian, C., Camacho, E., Kelly, J. E., Wang, H., Casadevall, A. & Stark, R. E., Solid-state NMR spectroscopy identifies three classes of lipids in Cryptococcus neoformans melanized cell walls and whole fungal cells. *J. Biol. Chem.* **2020,** *295*, 15083-15096.
2. Lamon, G. Lends, A., Valsecchi, I. Wong, S. S. W.; Duprès, V., Lafont, F., Tolchard, J., Schmitt, C., Mallet, A. & Grélard, A., Solid-state NMR molecular snapshots of Aspergillus fumigatus cell wall architecture during a conidial morphotype transition. *Proc. Natl. Acad. Sci. USA* **2023,** *120*, e2212003120.
3. Consolacion, Y., Oscar, B., Chien-Chang, S., Gayle, R., Michelle, M. & Joyce, F. R., Chemical constituents of Aglaia loheri. *Pharmacogn. J.* **2012,** *4*, 29-31.
4. Suttiarporn, P., Chumpolsri, W., Mahatheeranont, S., Luangkamin, S., Teepsawang, S. & Leardkamolkarn, V. Structures of phytosterols and triterpenoids with potential anti-cancer activity in bran of black non-glutinous rice. *Nutrients* **2015,** *7*, 1672-1687.
5. Tuckey, R. C., Nguyen, M. N., Chen, J., Slominski, A. T., Baldisseri, D. M., Tieu, E. W., Zjawiony, J. K. & Li, W., Human cytochrome P450scc (CYP11A1) catalyzes epoxide formation with ergosterol. *Drug Metab. Dispos.* **2012,** *40*, 436-444.
6. Rodríguez-Pupo, E.C.; Pérez-Llano, Y.; Tinoco-Valencia, J.R.; Sánchez, N.S.; Padilla-Garfias, F.; Calahorra, M.; Sánchez, N.d.C.; Sánchez-Reyes, A.; Rodríguez-Hernández, M.d.R.; Peña, A.; Sánchez, O.; Aguirre, J.; Batista-García, R.A.; Folch-Mallol, J.L.; Sánchez-Carbente, M.d.R. Osmolyte Signatures for the Protection of *Aspergillus sydowii* Cells under Halophilic Conditions and Osmotic Shock. *J. Fungi* **2021**, *7*, 414.
7. Shim, J. H., Sung, K. J., Cho, M. C., Choi, W. A., Yang, Y., Lim, J. S. & Yoon, D. Y., Antitumor effect of soluble beta-1,3-glucan from Agrobacterium sp. R259 KCTC 1019. *J. Microbiol. Biotechnol.* **2007,** *17*, 1513-1520.
8. Fernando, L. D., Dickwella Widanage, M. C., Penfield, J., Lipton, A. S., Washton, N., Latgé, J.-P., Wang, P.; Zhang, L. & Wang, T., Structural polymorphism of chitin and chitosan in fungal cell walls from solid-state NMR and principal component analysis. *Front. Mol. Biosci.* **2021,** *8*, 727053.
9. Bhanja, S. K., Rut, D., Patra, P., Sen, I. K., Nandan, C. K. & Islam, S. S., Water-insoluble glucans from the edible fungus Ramaria botrytis. *Bioact. Carbohydr. Diet. Fibre* **2014,** *3*, 52-58.
10. Chakraborty, A., Fernando, L. D., Fang, W., Dickwella Widanage, M. C., Wei, P., Jin, C., Fontaine, T., Latgé, J.-P. & Wang, T., A molecular vision of fungal cell wall organization by functional genomics and solid-state NMR. *Nat. Commun.* **2021,** *12*, 6346.
11. Fritzsching, K., Yang, Y., Schmidt-Rohr, K. & Hong, M., Practical use of chemical shift databases for protein solid-state NMR: 2D chemical shift maps and amino-acid assignment with secondary-structure information. *J. Biomol. NMR* **2013,** *56*, 155-167.
